# Analysis of Water Distribution and Muscle Quality of Silver Carp (*Hypophthalmichthys molitrix*) Chunks Based on Electron-Beam Irradiation

**DOI:** 10.3390/foods11192963

**Published:** 2022-09-22

**Authors:** Hai-Lan Li, Mei-Jin Li, Qing Zhao, Jia-Jun Huang, Xiao-Yan Zu

**Affiliations:** 1 Institute of Agricultural Products Processing and Nuclear Agricultural Technology, Hubei Academy of Agricultural Sciences, Wuhan 430064, China; 2 Key Laboratory of Cold Chain Logistics Technology for Agro-Product, Ministry of Agriculture and Rural Affairs, Wuhan 430064, China; 3 College of Bioengineering and Food, Hubei University of Technology, Wuhan 430068, China

**Keywords:** electron-beam irradiation, silver carp chunks, water distribution, muscle quality

## Abstract

Electron-beam irradiation (EBI) is an efficient, safe, and nonthermal sterilization technique that is extensively used in food preservation research. Here we report the effects of different EBI doses (0, 4, 8 kGy) and preservation temperatures (room temperature [RT], 4 °C) on the muscle water distribution and muscle quality indices of silver carp chunks (SCCs). The highest entrapped water content was found in the 4-kGy-irradiated/4-°C-stored samples. The expressible moisture content (EMC) of the SCCs increased with increasing irradiation dose and was significantly lower in the RT group than in the 4 °C group. The irradiation dose and preservation temperature had no significant effect on the moisture content, whiteness value and protein content of SCCs (*p* > 0.05). When the irradiation dose reached 8 kGy, AV value, POV value and TVB value were significantly increased (*p* < 0.05). The myofibrillar protein content and actomyosin content of the SCCs in the 4 °C group was higher than that of the specimens in the RT group by 0.29–0.98 mg/mL (*p* < 0.05) and 36.21–296.58 μg/mL (*p* < 0.05), respectively. Overall, EBI treatment (4 kGy) and low-temperature preservation (4 °C) helped retain the muscle water content of the SCCs and preserve their quality, thereby endorsing the EBI treatment of silver carp products.

## 1. Introduction

Silver carp or white carp (*Hypophthalmichthys molitrix*), which is a fish species that belongs to the Cyprinidae family in the order Clupeiformes is a globally abundant freshwater resource [1]. Silver carp is popular among consumers for being nutritious, tender, and inexpensive [2]. However, the flesh of silver carp is susceptible to microbial and endogenous enzymatic activities as well as biochemical reactions, which can lead to spoilage during storage as well as the production of volatile basic nitrogen and free amino acids [3]. In addition to traditional processing and preservation methods such as curing, refrigeration, freezing, and heat treatment, advanced techniques such as modified atmosphere packaging [4], ultrahigh-pressure sterilization [5], snap freezing [6], and irradiation [7] have been adopted to maintain the nutritional quality and edible value of silver carp flesh. Among these methods, ultrahigh-pressure sterilization and snap freezing are cost ineffective owing to their requirement of expensive equipment and high costs, which hinder their widespread adoption. Moreover, the utilization of the modified atmosphere technology is limited by the variation in the gas components in the modified atmosphere system from product to product and the stringent requirements for packaging materials.

Irradiation is a safe and effective cold-sterilization technique, with electron-beam irradiation (EBI) being a particularly noteworthy food-decontamination technology that is cost effective, easy to manipulate, and contamination free [8,9]. Electron accelerator produced high energy electron beams can sterilize food products and extend their shelf life while maintaining their original quality and flavor [10]. As an advanced technique used in food preservation, EBI has been found to effectively reduce or eliminate nitrates and nitrites in cured meat [11]. H. Yu et al. found that EBI treatment at 10 kGy significantly reduced the total volatile basic nitrogen content in cod and increased its water and ash contents [12]. H. Guo et al. studied the effects of EBI on the volatile flavor substances of salmon fillets and showed that the best sensory flavor of fish flesh was achieved with 1 kGy irradiation treatment [13]. Q. Yu et al. found that EBI had no effect on the pH of shrimp and that higher irradiation doses resulted in fish with more odorous volatiles [14]. Recent studies on the EBI of aquatic products have primarily focused on changes in their physicochemical properties and sensory flavor; however, investigations related to the influence of EBI on the water distribution in silver carp flesh have been rarely reported.

Low-field nuclear magnetic resonance (LF-NMR) is a rapid, accurate, and nondestructive analytical detection technique. Using this method, the flow and distribution of hydrogen protons in tissues of food products can be determined by assessing the relaxation properties of hydrogen protons in a constant magnetic field, which reflect the water content and migration processes in samples [15]. LF-NMR has been applied to various sea products, including oysters [16], hake [17], and sea cucumbers [18]. However, studies focusing on the LF-NMR analysis of freshwater products are relatively scarce.

Therefore, this study took SSCs as the research object to explore the effects of different preservation temperatures (room temperature [RT], 4 °C) and different EBI doses (0, 4, 8 kGy) on the water distribution of SSCs, as well as analyzing the correlation between EBI, preservation temperature and muscle quality of SSCs. The results reported herein are anticipated to enable improvements in the processing and quality control of silver carp products through EBI treatment.

## 2. Materials and Methods

### 2.1. Materials

Analytical-grade chemicals including hydrochloric acid, boric acid, petroleum ether, isopropyl alcohol, trichloromethane, sodium hydroxide, anhydrous sodium sulfate, and TBA were purchased from Sinopharm Group Chemical Reagent Co., Ltd. (Shanghai, China). A Bradford Protein Assay Kit (P0006) was purchased from Shanghai Beyotime Biotechnology Co., Ltd. (Shanghai, China), and a total protein assay kit (A045-4-2) was procured from Nanjing Jiancheng Bioengineering Institute.

### 2.2. Sample Preparation and Processing

Fresh silver carp (*n* = 20) with weights and body lengths of 2.0 ± 0.2 kg and 54 ± 2.3 cm, respectively, were purchased from Wushang Supermarket (Agricultural Science City Store), Hongshan District, Wuhan, China. The silver carp was washed, and its head, tail, scales, internal organs, and bones were removed. Subsequently, the flesh was cut along the backbone into chunks weighing 20 ± 2.0 g. These portions were placed individually in low-density-polyethylene bags, sealed with a sealing machine, and then heated in boiling water for 2 min to cook the flesh to medium-rare. The fish samples were cooled, skinned, and randomly divided into two groups with equal sample sizes based on temperature—room temperature (RT; 25 °C) and 4 °C—with the 4 °C group samples being stored in iceboxes. The samples from both groups were separated into three subgroups, which were then subjected to EBI treatment at doses of 0, 4, and 8 kGy, respectively, at Wuhan Aibang High Energy Technology Co., Ltd. (Wuhan, China). After the EBI treatment, the samples were placed in iceboxes and then stored at RT or 4 °C. Subsequently, the samples were experimentally investigated to determine relevant indices. The actual doses received by the samples were calibrated with low- and high-dose-range silver dichromate dosimeters containing 0.35 and 2.5 mmol/L silver dichromate, respectively [19]. The dosimeters monitored the irradiation of the samples and were sent to the Irradiation Engineering Center of Hubei Province; the actual absorbed doses of the three subgroups were determined to be 0, 4.25, and 7.84 kGy, respectively.

### 2.3. Determination of Water Distribution

LF-NMR and magnetic resonance imaging (MRI) analyses were performed using a slightly modified version of the method described by L. Wang et al. [20]. The SCCs were cut into 1.8 × 1.8 × 1.8 cm^3^ pieces, padded dry, and placed in MRI-compatible test tubes. The distributions of bound, entrapped, and free water were monitored using an NMR analyzer (NMI20-025V-I, Suzhou Niumag Analytical Instrument Co., Ltd., Suzhou, China). The water in the SCCs could be classified into the following three categories based on how tight the water was bound to the tissues: bound water that is tightly tethered to muscle macromolecules (T_2b_), entrapped water retained by myofibrils (T_21_), and free water held in interfascicular space and by sarcoplasmic proteins (T_22_) [21]. The following parameters were used for the T_2_ measurements: resonant frequency (SF), 20 MHz; 90° pulse width (P1), 8 μs; 180° pulse width (P_2_), 16.48 μs; spectral width (SW), 100 kHz; waiting time (TW), 1000 ms; econ time (TE), 1 ms; echo number (NECH), 700; and repetitive scans number (NS), 4. The sample signals were acquired using the CPMG sequence and NMR analysis software. The inverse transformation was performed using SIRT100000 to obtain the relaxation time and peak area for each water type. Additionally, the pseudo-color maps of the water molecules were constructed based on the analysis of water proton densities.

### 2.4. Determination of Moisture Content and Expressible Moisture Content (EMC)

The moisture content was determined using the method described by Shi et al. with slight modifications [22]. Clean aluminum weighing bottles were dried in an oven at 55 °C for 1 h (DHG-9203A, Shanghai Yi Heng Co., Ltd., Shanghai, China), removed, covered, and then transferred to a desiccator to cool for 20 min before being weighed. The drying and cooling processes were repeated until a constant weight was achieved, which was based on the difference between two successive weights not exceeding 2 mg. The SCCs were minced, weighed, and placed in the prepared weighing bottles. The aforementioned drying, cooling, and weighing processes were repeated, and the final constant weights were recorded. The water content was calculated using the following equation:

(1) $$\mathrm{Moisture}\;\mathrm{content}\;(g/100\;g)=\frac{m_{1}-m_{2}}{m_{1}}\;\times \;100,$$

where *m*_1_ is the weight of the SCCs immediately after mincing (g), *m*_2_ is the constant weight of the minced SCCs (g), and 100 is the conversion factor.

The EMC was determined using a slightly modified version of the method described by Jiao et al. [23]. Briefly, the SCCs were weighed, wrapped with filter paper, and then centrifuged (TGL-24MC, Changsha Pingfan Instrument Co., Ltd., Changsha, China) at 2000 rpm for 15 min. The centrifuged samples were weighed, and the EMC was calculated using the following equation:

(2) $$\mathrm{EMC}\;(\%)=\frac{m_{1\;}-m_{2}}{m_{1}}\;\times \;100\%,$$

where *m*_1_ and *m*_2_ (g) are the masses of the SCCs before and after the centrifugation, respectively.

### 2.5. Determination of Whiteness and pH Values

As an important appearance related indicator of aquatic products, whiteness value directly affects the initial assessment of these products by consumers [24]. Based on the method described by Gulcan et al. [25], the brightness (L*), redness (a*), and yellowness (b*) values of the cut surface of SCCs were measured using a colorimeter (CR-400, Konica Minolta, Japan), and the whiteness values were calculated thereafter as follows:

(3) $$\mathrm{Whiteness}=100\;-\;\sqrt{{\left(100\;-\;L*\right)}^{2}+{a*}^{2}+{b*}^{2}},$$

pH is an important indicator of the quality and freshness of aquatic products [26]. For the pH analysis, 4 g of an SCC sample was mashed and mixed with 40 mL of distilled water. The mixture was thoroughly agitated and left to stand for 20 min before being filtered. The pH value of the collected filtrate was then determined using a pH meter (Mettler-Toledo Instruments Co., Ltd., Shanghai, China).

### 2.6. Determination of AVs, Peroxide Values (POVs), and TBA Values

AVs, POVs, and TBA values are important indicators for evaluating the extent of lipid hydrolysis as well as primary and secondary lipid oxidation [27]. AVs were determined according to the GB5009.229-2016 standard and the method reported by Wei et al. [28]. The AVs of the SCCs were calculated as follows:

(4) $$\mathrm{AV}\;(\mathrm{mg}/g)=\frac{\left({V\;-\;V}_{0}\right)\;\times \;0.01\;\times \;56.1}{m},$$

where *V* and *V*_0_ (mL) are the volumes of the standard titration solution consumed for determination of the samples and the corresponding blanks, respectively; 0.01 is the molar concentration of a potassium-hydroxide standard solution (mol/L); 56.1 is the molar mass of potassium hydroxide (g/mol); and m is the weight of the SCC sample (g).

POVs were determined according to the GB5009.227-2016 standard and the method reported by Wang et al. [29]. The POVs of the SCCs were calculated as follows:

(5) $$\mathrm{POV}\;(g/100\;g)=\frac{\left({V\;-\;V}_{0}\right)\times \;0.01\;\times \;0.1269}{m}\;\times \;100,$$

where *V* and *V*_0_ (mL) are the volumes of the sodium thiosulfate standard solution consumed by the samples and blanks, respectively; 0.01 is the molar concentration of the sodium-thiosulfate standard solution (mol/L); 0.1269 is the mass of elementary iodine equivalent to 1 mL of the sodium-thiosulfate standard titration solution [*c*(Na_2_S_2_O_3_) = 1.000 mol/L]; m is the weight of the SCC sample (g); and 100 is the conversion factor.

The TBA values were determined using a slightly modified version of the method described by Salih et al. [30]. Five grams of an SCC sample was placed in a centrifuge tube and mixed with 25 mL of a trichloroacetic acid solution (20% volume fraction). The mixture was uniformly stirred and left to stand for 1 h before being centrifuged at 2000 rpm for 10 min and then filtered. Distilled water was added to the collected filtrate to achieve a final volume of 50 mL. A portion of this filtrate (5 mL) was mixed with 5 mL of TBA solution (0.02 mol/L) and reacted in a boiling water bath for 20 min. After cooling, the mixture was subjected to absorbance (A) analysis at 532 nm using a spectrophotometer (UH5300, Hitachi Co., Ltd., Tokyo, Japan), and the TBA values were calculated as follows:

TBA (mg/100 g) = *A* × 7.8, (6)

where *A* is the absorbance of the solution measured at 532 nm, and 7.8 is a constant.

### 2.7. Determination of Total Protein Content, Myofibrillar Protein Content, and Actomyosin Content

The total protein contents were determined according to the GB5009.5-2016 standard and the method reported by Yang et al. [31]; the corresponding values of the SCCs were obtained as follows:

(7) $$\mathrm{Protein}\;\mathrm{content}\;(g/100\;g)=\frac{\left(V_{1\;}-{\;V}_{2}\right)\;\times \;0.05\;\times \;0.0140}{m\;\times \;10∕100}\;\times \;F\;\times \;100,$$

where *V*_1_ and *V*_2_ are the volumes of the hydrochloric-acid standard titrant consumed by the specimens and blanks, respectively; 0.05 is the concentration of the HCl standard titrant (mol/L); 0.0140 is the mass of elementary nitrogen equivalent to 1 mL of the HCl standard titrant [*c*(HCL) = 1.000 mol/L], *m* is the weight of the SCC sample (g); 10 is the volume of digested sample used for titration (mL); *F* is the nitrogen-to-protein conversion factor; and 100 is the general conversion factor.

The content of myofibrillar protein (W/V) were determined using the method described by Benjakul et al. (1997) with slight modifications [32]. One gram of an SCC sample was mixed with 10 mL of precooled 0.1 mol/L KCl solution, followed by homogenization at 10,000 rpm for 1 min. The dispersion was then centrifuged at 10,000 rpm for 20 min at 4 °C, and the supernatant was discarded. The resulting precipitate was resuspended in a 0.6 mol/L KCl solution with a volume 8× its original value and homogenized for 1 min (XHF-D, Ningbo Xinzhi Co., Ltd., Ningbo, China). The mixture was then left to stand at 4 °C for 1 h and centrifuged thereafter at 12,000 rpm for 30 min at 4 °C. The supernatant was collected, and the myofibrillar protein content of the samples was determined using a quantitative protein assay kit.

The actomyosin contents were determined using the method described by Zhou et al. with slight modifications [33]. Two grams of an SCC sample was minced and mixed with 10 mL of precooled 0.6 mol/L KCl solution. The mixture was homogenized at 10,000 rpm for 30 s and then centrifuged at 5000 rpm for 30 min at 4 °C. The supernatant was collected and diluted in precooled distilled water with a volume 3× the original value. The diluted supernatant was further centrifuged at 5000 rpm for 20 min at 4 °C, and the precipitate was collected and resuspended in a precooled 1.2 mol/L KCL solution with equal volume. The resulting mixture was blended using a magnetic stirrer (DF-101S, Wuhan Ke’er Instrument Co., Ltd., Wuhan, China) for 30 min and then centrifuged at 5000 rpm for 20 min at 4 °C. The supernatant was collected, and the actomyosin content was determined using a quantitative protein assay kit.

### 2.8. Data Analysis

Origin 2019 software was used for graphically visualizing the results, whereas SPSS 26.0 and Microsoft Office Excel 2016 were used for statistical data analysis. The results are presented as mean ± standard deviation (Mean ± SD). Analysis of variance (ANOVA) was used for significance analysis, with a P-value less than 0.05 (*p* ≤ 0.05) considered to be statistically significant.

## 3. Results and Discussion

### 3.1. Effects of Different EBI Doses on the Water Distribution in SCCs

**Figure 1:**
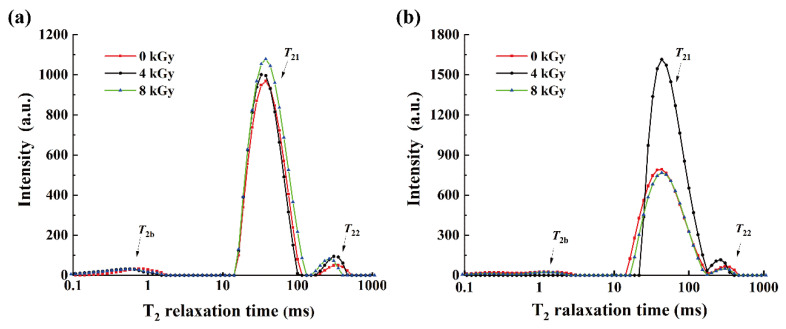
Effect of irradiation dose on moisture distribution of silver carp chunks stored at room temperature (**a**) and 4 °C (**b**).

**Figure 2:**
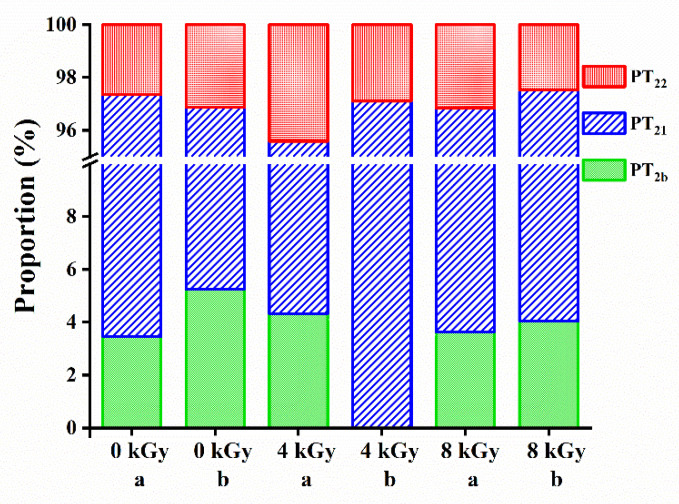
Diagram of relative moisture content of silver carp chunks at different irradiation doses stored at room temperature (**a**) and 4 °C (**b**).

The peak areas in T_2_ spectrums reflect the relative water content of the SCCs in the corresponding states. As shown in Figure 1a and Figure 2b, the peak area of the water content T_2b_ in samples that received the same dose of irradiation treatment was lower in the 4 °C group than in the RT group (*p* < 0.05), whereas T_21_ showed a rightward shift. These results indicate that low temperatures could promote the transformation of bound water to entrapped and free water, which may lead to a weakened water-binding capability of silver carp flesh and, consequently, an increase in EMC (Figure 4b). Compared with that in the unirradiated group, T_22_ of samples in both the irradiated groups (RT and 4 °C) showed a leftward shift and an increase in the peak areas, indicating that the irradiation reduced the loss of free water from the SCCs and transformed it into entrapped water. This was possibly caused by the change in the state of free water through the EBI-induced weakening of hydrogen bonds [34]. For the unirradiated samples, the sum of the T_21_ and T_22_ peak areas of samples in the 4 °C group was greater than that of specimens in the RT group, suggesting that low temperatures could also moderately reduce the drip loss of silver carp flesh. Figure 1b and Figure 2 show that the signal from the bound water disappeared in the 4-kGy-irradiated/4-°C-stored group. This disappearance was probably due to the large peak area and high peak intensity of the signal from the entrapped water as well as the conversion of bound and free water to entrapped water, which led to a further decrease in the originally low bound-water proportion. The peak area of the signal representing entrapped water increased with increasing irradiation dose at RT. However, at 4 °C, the entrapped-water content was the highest in the 4-kGy-irradiated samples whereas it was reduced in the 8-kGy-irradiated specimens. In conclusion, low temperatures and irradiation could both reduce the drip loss of silver carp flesh, and the high content of entrapped water in the 4-kGy-irradiated/4-°C-stored group suggested that irradiation at 4 kGy and preservation at 4 °C improved the tenderness and quality of the silver carp flesh.

**Figure 3:**
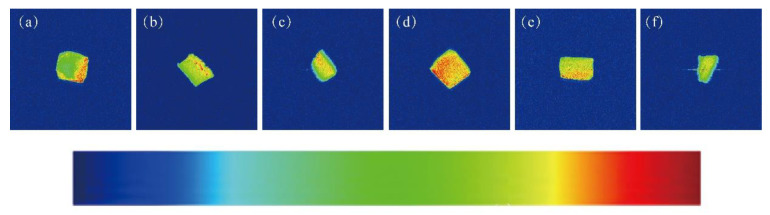
Pseudo-color maps of water molecular proton density of silver carp chunks with different treatment methods. (**a**) Room temperature, 0 kGy; (**b**) 4 °C, 0 kGy; (**c**) Room temperature, 4 kGy; (**d**) 4 °C, 4 kGy; (**e**) Room temperature, 8 kGy; (**f**) 4 °C, 8 kGy.

The pseudo-color maps of water proton density constructed under different treatment conditions are shown in Figure 3. These maps reflect the distribution of water, with the areas featuring strong signals presented in red and the signal-free zones indicated in blue. The brightness of an image increases with increasing proton density, indicating a higher water content in the sample and less drip loss [35]. As shown in Figure 3, the brightness of the proton density pseudo-color maps decreased in the following manner: (d) > (e) > (b) > (c) > (a) > (f); this is consistent with the results shown in Figure 1a,b. Among the SCCs stored at RT, the samples of the irradiated group were darker than those in the unirradiated batch, and the red signals in the maps intensified with increasing EBI dose. The signal intensity of the 4-kGy-irradiated/4-°C-stored samples was the strongest and higher than that of the RT stored equivalents. This may be because T_22_ showed a leftward shift most obviously under this condition, which reduced the loss of free water to the greatest extent. These results indicate that EBI could effectively reduce the drip loss of silver carp flesh and, in essence, inhibit the loss and diffusion of water in SCCs. Moreover, the preservation temperature of 4 °C and EBI dose of 4 kGy were effective in maintaining the quality of the silver carp product.

### 3.2. Effects of Different EBI Doses on the Moisture Content and EMC Loss of SCCs

**Figure 4:**
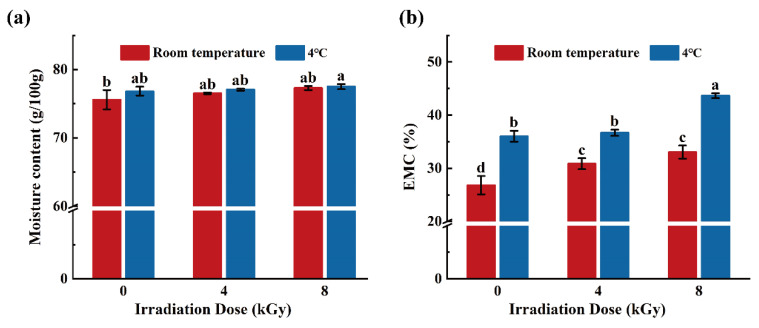
Effect of different doses of electron beam irradiation on moisture content (**a**) and EMC (**b**) of silver carp chunks. Different lowercase letters indicate significant difference among groups (*p* < 0.05).

All As shown in Figure 4a, the moisture content of SCC samples in the 4 °C group was not significantly different from that of the RT group (*p* > 0.05). Moreover, the moisture contents of the SCC samples treated at the same temperature but irradiated with different doses were not significantly different from those of the unirradiated samples (*p* > 0.05). Yang et al. showed that the moisture content of EBI-treated vacuum-packed Atlantic salmon fillets was not significantly correlated with the irradiation dose, which is consistent with the findings reported herein [36]. 

Figure 4b shows that the rate of EMC loss of the centrifuged SCC samples was significantly lower in the RT group than in the 4 °C group (*p* < 0.05). Under ambient conditions, the EMC loss rates of the samples in the irradiated groups were significantly higher than those of the unirradiated samples (*p* < 0.05), whereas no significant difference was observed between the irradiated groups (*p* > 0.05). For the samples stored at 4 °C, the rates of EMC loss were not significantly different between the 4-kGy-irradiated and unirradiated samples (*p* > 0.05), whereas the 8-kGy-irradiated samples showed significantly higher values than those of the 4-kGy-irradiated counterparts (*p* < 0.05). The EMC was used to determine the water-holding capacity of the irradiated SCCs by exploiting the inverse proportionality between these parameters [37]. These results suggest that low temperatures and high irradiation doses may lead to a reduced water holding capacity and an increased EMC loss rate of SCCs.

### 3.3. Effects of Different EBI Doses on the Whiteness and pH Values of SCCs

**Figure 5:**
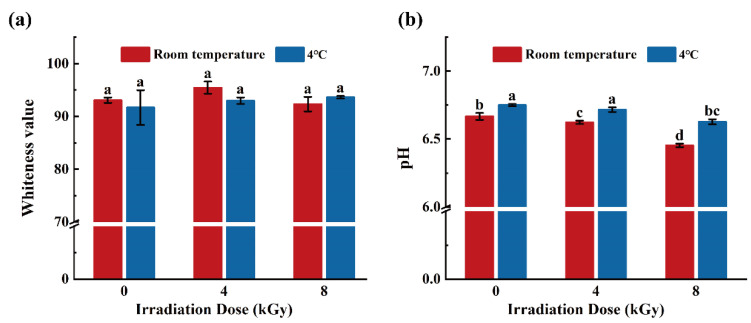
Effect of different doses of electron beam irradiation on whiteness value (**a**) and pH (**b**) of silver carp chunks. Different lowercase letters indicate significant difference among groups (*p* < 0.05).

As shown in Figure 5a, the effect of EBI on the whiteness value of the SCC samples was not significant at both RT and 4 °C (*p* > 0.05), indicating that EBI did not cause significant color related changes in the fish flesh. Zhang et al. conducted EBI treatment of vacuum-packed grass carp surimi and found that the whiteness values did not differ significantly among irradiated groups after EBI on the zeroth day of storage (*p* > 0.05), which is similar to the results reported herein [38].

The pH values of the SCCs stored at RT decreased significantly (*p* < 0.05) with increasing irradiation dose (Figure 5b). This may be because irradiation can moderately facilitate the breakdown of muscle glycogen, which produces acids such as ATP, lactate, and phosphocreatine, thereby leading to the decrease in sample pH and, consequently, accelerated food spoilage [39]. Ham. Y.-K et al. also found that the pH value of cooked pork sausages irradiated with electron-beam decreased with increasing absorbed dose level (*p* < 0.05) [40]. The pH values of the SCC samples were significantly higher in the 4 °C group than in the RT group (*p* < 0.05). Among the samples in the 4 °C group, the pH values of 4-kGy-irradiated samples were not significantly different from the unirradiated counterparts (*p* > 0.05). These results indicate that storage at 4 °C and EBI treatment at 4 kGy assisted in maintaining the original pH of the SCCs, and the low temperature could reduce the EBI-induced quality loss of SCCs.

### 3.4. Effects of Different EBI Doses on AVs, POVs, and TBA Values of SCCs

**Figure 6:**
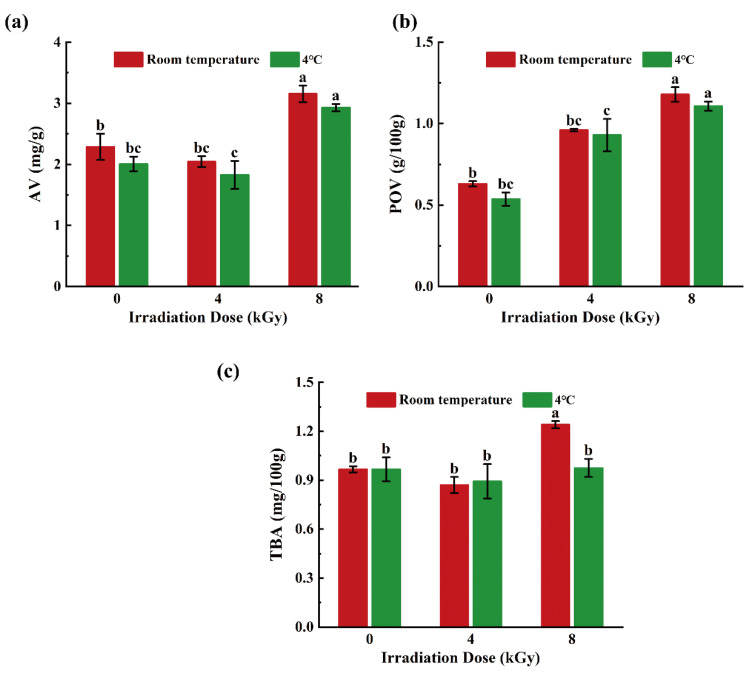
Effects of different doses of electron beam irradiation on TBA value (**a**), AV value (**b**) and POV value (**c**) of silver carp chunks. Different lowercase letters indicate significant difference among groups (*p* < 0.05).

Temperature had minor effects on the AVs and POVs of the samples (Figure 6a,b). No significant difference (*p* > 0.05) was found in the AVs and POVs between the samples treated with the same irradiation dose but at different temperatures. Overall, the AVs and POVs of samples in both temperature groups increased with increasing irradiation dose. However, the AVs and POVs of the 4-kGy-irradiated samples were not significantly different from those of the unirradiated specimens (*p* > 0.05), but were significantly lower than those of the 8-kGy-irradiated counterparts (*p* < 0.05). Oxidation of fats and oils yields unsaturated fatty acids, which can be further oxidized upon exposure to light and heat, resulting in the production of organic acids and, consequently, food rancidity; moreover, higher AVs indicate a greater degree of fat and oil oxidation [41]. The 4-kGy-irradiation treatment minimally affected the AVs of the SCCs, whereas the 8-kGy-irradiation treatment accelerated their rancidification, resulting in higher AVs. The elevated POVs of the 8-kGy-irradiated SCCs may be due to the expedited lipid oxidation induced by the free radicals in the tissues that are generated by high-dose irradiation [42,43].

The TBA values of the 8-kGy-irradiated samples in the RT group were higher than those of the 4-kGy-irradiated and unirradiated counterparts (Figure 6c; *p* < 0.05). However, in the 4 °C group, the TBA values of the 8-kGy-irradiated samples were not significantly different from those of the 4-kGy-irradiated equivalents (*p* > 0.05). This indicates that high dose EBI under ambient conditions could promote the decomposition of unsaturated fatty acids and accelerate lipid oxidation, whereas low-temperature preservation could delay lipid oxidation and thus counteract the adverse effects of irradiation. The TBA values and POVs of EBI treated pork jerky have been found to increase in an EBI-dose-dependent manner [44]. EBI can catalyze the production of free radicals in fish products and accelerate lipid oxidation, triggering a free-radical chain reaction that increases the TBA values [45], which is similar to the mechanism by which EBI increases the POVs.

### 3.5. Effects of Different EBI Doses on Total Protein Content, Myofibrillar Protein Content, and Actomyosin Content

**Figure 7:**
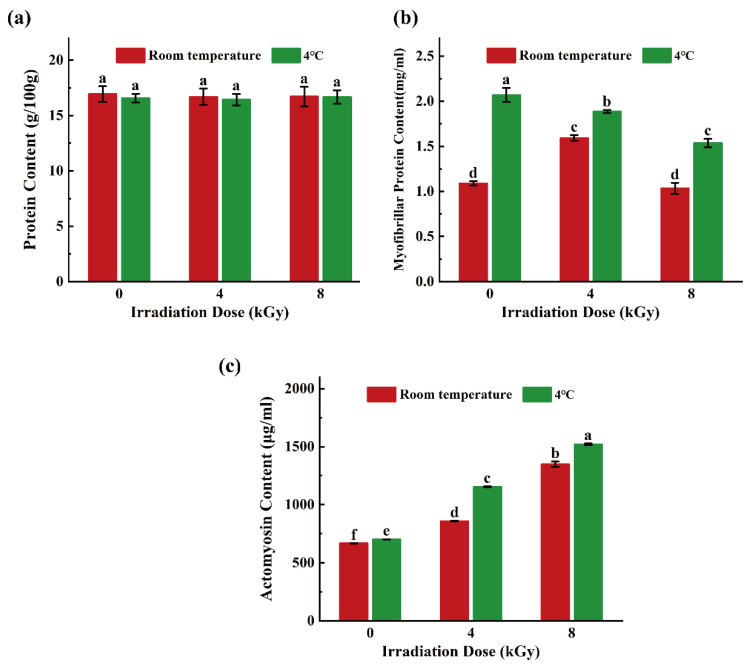
Effects of different doses of electron beam irradiation on protein content (**a**), myofibrillar protein content (**b**) and actomyosin content (**c**) of silver carp chunks. Different lowercase letters indicate significant difference among groups (*p* < 0.05).

Fish meat is a major source of animal protein for consumers owing to its high protein content [46]. The total protein contents of samples in all groups ranged from 16 g/100 g to 17 g/100 g, and the variation between all samples was not significant (*p* > 0.05; Figure 7a). The aforementioned findings are consistent with those of Fallah et al. who found that irradiation minimally affected the crude protein content in camel meat [47].

Myofibrillar protein constitutes the myofibrils in muscles and directly affects the juiciness, texture, and elasticity of meat products. The myofibrillar protein content in the 4-kGy-irradiated/RT-stored samples was 1.59 mg/mL (Figure 7b), which was a 31.64% increase over that of the unirradiated samples (*p* < 0.05); moreover, the difference between the 8-kGy-irradiated and unirradiated samples was not significant (*p* > 0.05). This indicated that the 4 kGy EBI treatment and RT storage helped improve the elasticity and texture of the SCCs. Additionally, the myofibrillar protein content of the SCC samples in the 4 °C group was higher than that of the specimens in the RT group by 0.29–0.98 mg/mL (*p* < 0.05), possibly due to the low-temperature-induced inhibition of myofibrillar protein oxidation [48].

The actomyosin contents of the SCC samples in both temperature groups increased in an EMI-dose-dependent manner (Figure 7c), with the actomyosin contents of the irradiated samples being significantly higher than those of the unirradiated specimens (*p* < 0.05). Additionally, the actomyosin content of the samples in the 4 °C group was significantly higher than that of the samples in the RT group and ranged from 36.21 to 296.58 μg/mL (*p* < 0.05). Actomyosin is the main component of myofibrillar protein, and the denaturation and loss of myosin are considered valid indicators of quality loss of fish meat [49]. These results suggest that low-temperature preservation (4 °C) and EBI treatment could inhibit the denaturation and decomposition of actomyosin and effectively improve the quality of fish protein.

## 4. Conclusions

In this study, the effects of different preservation temperatures and EBI doses on the quality of SCCs were investigated. The results showed that low preservation temperatures and 4 kGy EBI treatment could prevent the partial free water loss in the meat of silver carp and consequently improve the tenderness and quality of the meat. The centrifugation induced EMC loss of the SCCs was significantly and positively correlated with the irradiation dose, and the water holding capacity of the samples in the 4 °C group was lower than that of the samples in the RT group. Low preservation temperature prevented the loss of myofibrillar protein and actomyosin, and EBI effectively inhibited actomyosin degeneration. Moreover, the myofibrillar protein content tended to decrease with increasing irradiation dose. When the irradiation dose reached 8 kGy, the oxidation of silver carp was accelerated and the pH value was increased. Overall, preservation at 4 °C and EBI treatment at 4 kGy were determined to be the optimal EBI processing conditions for SCCs. The findings reported herein are in support of the irradiation-based preservation of silver carp products.

## Data Availability

The data showed in this study are contained within the article.
